# Impact of a dedicated trauma desk in ambulance control on the identification of major trauma in Scotland

**DOI:** 10.1186/cc13256

**Published:** 2014-03-17

**Authors:** J Bruce, A Parker, M Donald, M Esposito, L Curatolo, A Kennedy, K Simpson, C Morton, J Cormack, M Austin

**Affiliations:** 1 St Johns Hospital, Livingston, UK; 2 Scottish Ambulance Service, Edinburgh, UK; 3 Ninewells Hospital, Dundee, UK

## Introduction

In this study we determine the impact of a dedicated trauma desk on the identification of patients suffering from major trauma and on time to allocate critical care resources. Trauma is increasingly recognised as a serious global health problem and is the leading cause of death in those under the age of 45, accounting for 1,300 deaths in Scotland each year [1]. In addition, trauma causes considerable short-term and long-term morbidity and has personal, social and financial impact on the population [2]. Evidence and expert opinion strongly support the presence of appropriately trained, clinically active, personnel integrated and fundamental to the command and control structure in order to optimise the right resource being dispatched to the right patient at the right time.

## Methods

From October 2012 to April 2013 a trauma desk, staffed by experienced paramedics, was created in ambulance control with access to all emergency calls across Scotland. The operational hours of the desk were 08:00 to 18:00, 7 days a week. A customised database provided a data entry portal. Retrospective data were collated for comparison. Primary outcome measures were the number of emergency calls identified as potential major trauma and the time to allocation of critical care resources. Secondary outcome measures were the effects on stand-down rates of prehospital critical care teams.

## Results

There were 190 activations of critical care teams during the study period compared with 73 from the historical data from the same time frame in the previous year, representing a 160% increase in activations. The mean time from emergency call to allocation of critical care resource was 6 minutes compared with 19 minutes from historical data. The stand-down rate for prehospital critical care resource from emergency calls identified by trauma desk clinicians was 31.5% compared with 56% for those by nontrauma desk clinicians or dispatchers.

## Conclusion

A clinician focusing on major trauma in the ambulance control room improves activation times of prehospital critical care teams. The number of patients receiving life-saving and limb-saving interventions has substantially increased. The stand-down rate of teams following activation by the trauma desk is considerably reduced.
